# Biomechanical simulation of correcting primary unilateral cleft lip nasal deformity

**DOI:** 10.1371/journal.pone.0199964

**Published:** 2018-06-28

**Authors:** Hanyao Huang, Xiangyou Luo, Xu Cheng, Bing Shi, Jingtao Li

**Affiliations:** 1 State Key Laboratory of Oral Diseases, West China Hospital of Stomatology, Sichuan University, Chengdu, China; 2 National Clinical Research Center for Oral Diseases, West China Hospital of Stomatology, Sichuan University, Chengdu, China; 3 Dept. of Oral Maxillofacial Surgery, West China Hospital of Stomatology, Sichuan University, Chengdu, China; Ohio State University, UNITED STATES

## Abstract

For better outcomes of the primary correction of cleft lip nasal deformity, it is important to clarify the specific morphological and biomechanical consequences of major surgical maneuvers during cleft lip nose correction. In this study, a finite element model was established basing on the micro-MRI imaging of an infant specimen with unilateral complete cleft lip deformity. Alar base adduction was simulated as a medially-directed force on the lateral crus (F1); columella straightening was simulated as a laterally-directed force on the medial crus (F2); and nasal tip enhancement was simulated as an anteriorly-directed force on the intermediate crus (F3). The deformation and stress distribution consequent to each force vector or different force combinations were analyzed in details. Our biomechnical analyses suggested that W\when loaded alone, the three forces generated disparate morphological changes. The combination of different force loadings generated obviously different outcomes. F3 generated the most intensive stress when compared to F1 and F2. When F2 was loaded on top of F1-F3 combination, it further relieved nasal deviation without incurring significant increase in stress. Our simulation suggested that alar base adduction, columella straightening, and nasal tip elevation should all be included in a competent cleft lip nose correction.

## Introduction

**Fig 1:**
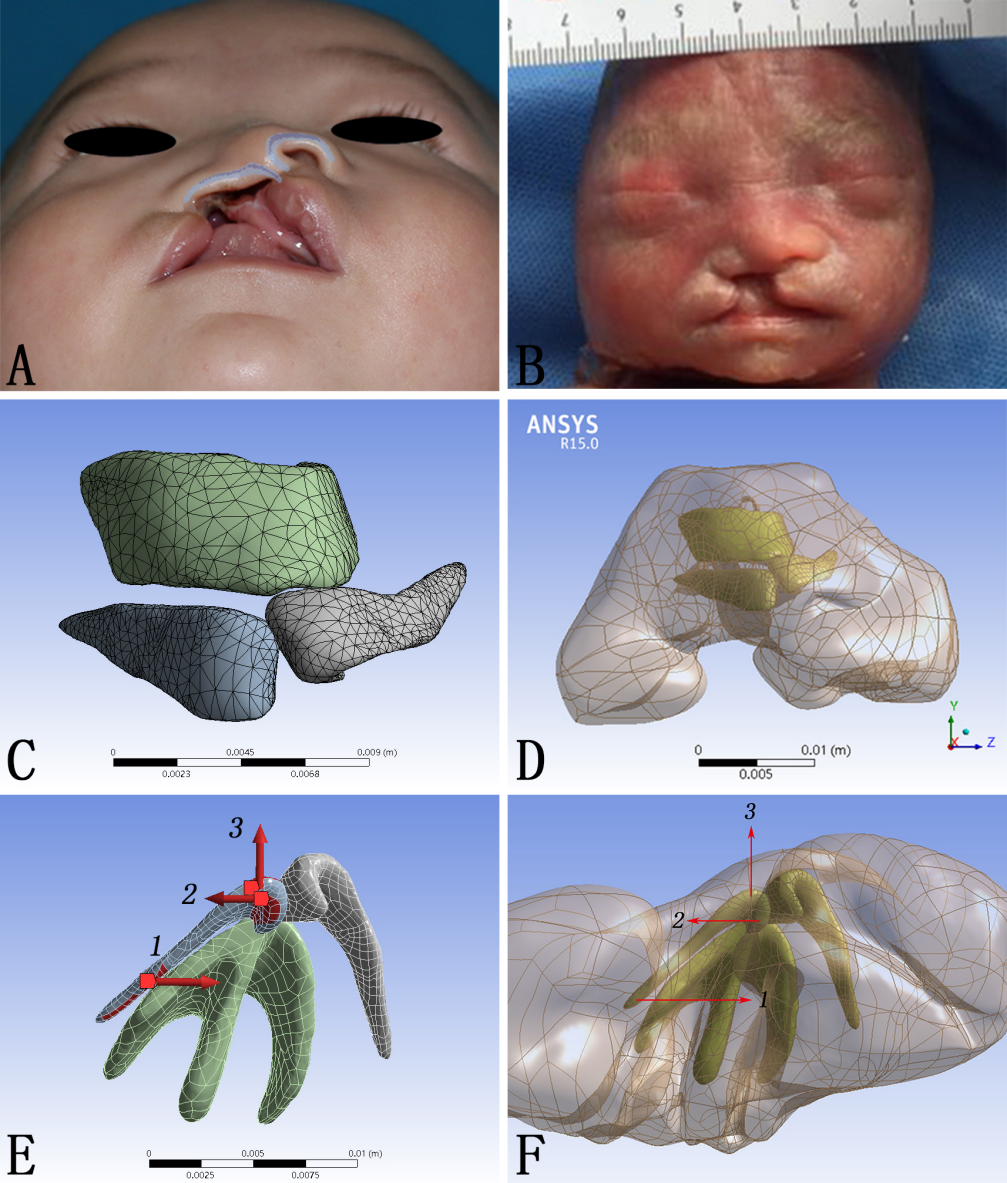
CAD model construction and vectors of force loadings. (A) Demonstration of a typical unilateral cleft lip nasal deformity. (B) Fetal specimen used for micro-MRI scanning. (C, D) The CAD model composed of both cartilage framework and skin envelope. (E, F) The directions of forces loaded on the alar cartilage.

Cleft lip deformity is one of the most common congenital anomalies in human, with an occurrence of 2/1000 in Asians [1]. Due to the displacement and hypoplasia of the alar cartilage framework in the cleft region, cleft lip nasal deformity is of some typical morphological characteristics, including the deviation of nasal tip and columella, displacement of nasal base, collapse of the alar dome, and discontinuity of the nasal sill (Fig 1A) [2]. Although contemporary surgical repair could constantly achieved satisfactory lip morphology, the accompanying nasal deformity persists. A major effort in modern cleft lip repair has been focusing on the nose correction [3]. Various combinations of surgical maneuvers have been applied to restore the anatomical nasal structure, but none is of constant success. Cleft lip nasal deformity remains a major challenge to surgeons and cleft management teams.

The fundamental principle of almost all surgical maneuvers is to release the pathologcial tethering and apply a corrective force on the nasal framework so as to restore the proper position of displaced tissues. From this perspective, an thorough understanding of the biomechanics in the cleft lip nasal deformity and its correction would be of great value to surgical modifications. Early physical simulations of the nasal structure, using paper board for example [4], illustrated correlations between the internal elements of the nasal cartilage framework, but failed to either mimic or quantify the morphological changes and stress. Finite-element scaling analysis, which can only quantify the morphological changes without stress, have been applied to study cleft nasal deformity with application of presurgical nasoalveolar molding [5]. Finite element analysis, on the other hand, serves as an ideal instrument to further our knowledge of the nasal biomechanics and has been successfully applied in many rhinoplasty scenarios [6–12].

Surgical correction of cleft lip nasal deformity is commonly composed of alar base adduction, columella straighterning, and nasal tip enhancement, all of which are basically force vectors generated on the nasal framework. The specific morphological consequence of each maneuvers, however, has not been explicitly illuminated. Furthermore, replase of the nasal deformity is common among cleft patients. Most of the relapse during growth was attributed to the tension generated during the surgery, which affects the nasal framework overtime. Thus, a comphrehensive understanding of the stress generated by each specific surgical maneuver would be of great value for the modification of corrective techniques. Both the morphological changes and stress distributions subsequent to the correction of cleft lip nasal deformity could be well analyzed with the aid of finite element model.

In this study, we set out to establish a finite element model of unilateral complete cleft lip nasal deformity, in which surgical corrections was simulated and their corresponding morphological deformation and stress distribution were depicted in details.

## Materials and methods

### Micro-MRI imaging

A nasolabial specimen from a Chinese induced labor with unilateral complete cleft lip was enrolled in December, 2016 (Fig 1B). For a clear scanning of this specimen, micro-MRI was applied (TE: 8.3 ms; FA: 180 deg; SI 0.40/0.40 mm; FOV 6.00 cm). DICOM-format images were exported into Mimics 15.0 (Mimics, Materialise, Belgium) for reconstruction.

### Nasal model construction

The unilateral complete cleft lip nasal deformity model was constructed according to the micro-MRI data and general anatomy of cleft lip nasal deformity. The nasal framework and the skin envelope made up the three dimensional model. The nasal framework consisted of two alar cartilages and the T-bar-shaped cartilaginous complex including the septal cartilage and the upper lateral cartilages (Fig 1C). The cartilage framework and the skin envelope were assembled through Pro/Engineer 5.0 (Pro/E) (PTC, Needham, MA, USA) (Fig 1D). The dimensions, including width, thickness, height and volume were shown in Table 1. Physical properties of the cartilages and the skin envelope were assigned according to published data as shown in Table 2 [8]. Finally, mesh generation of the model was performed using Workbench 15.0 (ANSYS Inc., Canonsburg, PA, USA) and exported in ASM format [13]. The definition of the meshwork was shown in details in Table 3.

**Table 1:** Definition of models for finite element analysis.

Geometry	Length X(x; mm)	Length Y(y; mm)	Length Z(z; mm)	Volume(v; mm^3^)
The skin envelope	19.46	22.84	35.05	5244.70
The left alar cartilage	9.88	3.82	6.07	22.44
The right alar cartilage	7.16	3.74	9.16	25.73
The T-bar-shaped cartilaginous complex	10.22	4.78	10.17	101.41

**Table 2:** Elastic properties of materials in the model for finite element analysis.

Materials [8]	Young’s modulus (MPa)	Density (kg/m^3^)	Poisson ratio
The skin envelope	0.5	980	0.33
The nasal cartilage	0.8	1080	0.15

**Table 3:** Definition of FE model in ANSYS Workbench.

Geometry	The skin envelope	The left alar cartilage	The right alar cartilage	The T-bar-shaped cartilaginous complex	total
Nodes	42066	5110	5088	5828	58092
Elements	25921	2715	2747	3146	34529

### Finite element simulation

The lateral margin of the skin envelope and the cranial margin of the cartilage framework where should attach to the piriform aperture were fixed to emulate the fixed connection between upper cartilage and maxilla. To simulate the functions of different forces caused by different operative methods secondary unilateral cleft lip nasal deformity, the forces were loaded on the alar cartilage at the cleft side in three different vectors: 1. Medially directed force on the lateral crus (F1); 2. Laterally directed force on the medial crus (F2); 3. Anteriorly directed force on the intermediate crus (F3) (Fig 1E and 1F). The direction and the magnitude (5N) of these three forces did not alter, but the combination of them changed: 1. Load F1; 2. Load F2; 3. Load F3; 4. Load F1 and F2 at the same time; 5. Load F1 and F3 at the same time; 6. Load F1, F2 and F3 at the same time. Static structural analysis was applied to calculate the total deformation (TD) and the equivalent von-mises stress (EQV).

The research protocol was censored and approved by the Ethic Committee of West China Hospital of Stomatology, Sichuan University (Approval No.WCHSIRB-D-2016-084R1). The picture of a patient with typical unilateral cleft lip nasal deformity (Fig 1A) was taken in November, 2016, and written informed consents were acquired from parents of this subject enrolled in this study (as outlined in PLOS consent form) to publish these case details. The specimen (Fig 1B) from one Chinese induced labor with unilateral complete cleft lip was enrolled in December, 2016, following the research protocol approved by the Ethic Committee. A signed consent from the parents was acquired at the time of induction to approve further scientific use of the specimen. Individual participant could not be identified during or after data collection.

## Results

### Accurate reconstruction of cleft lip nasal cartilage with Micro-MRI scanning data

**Fig 2:**
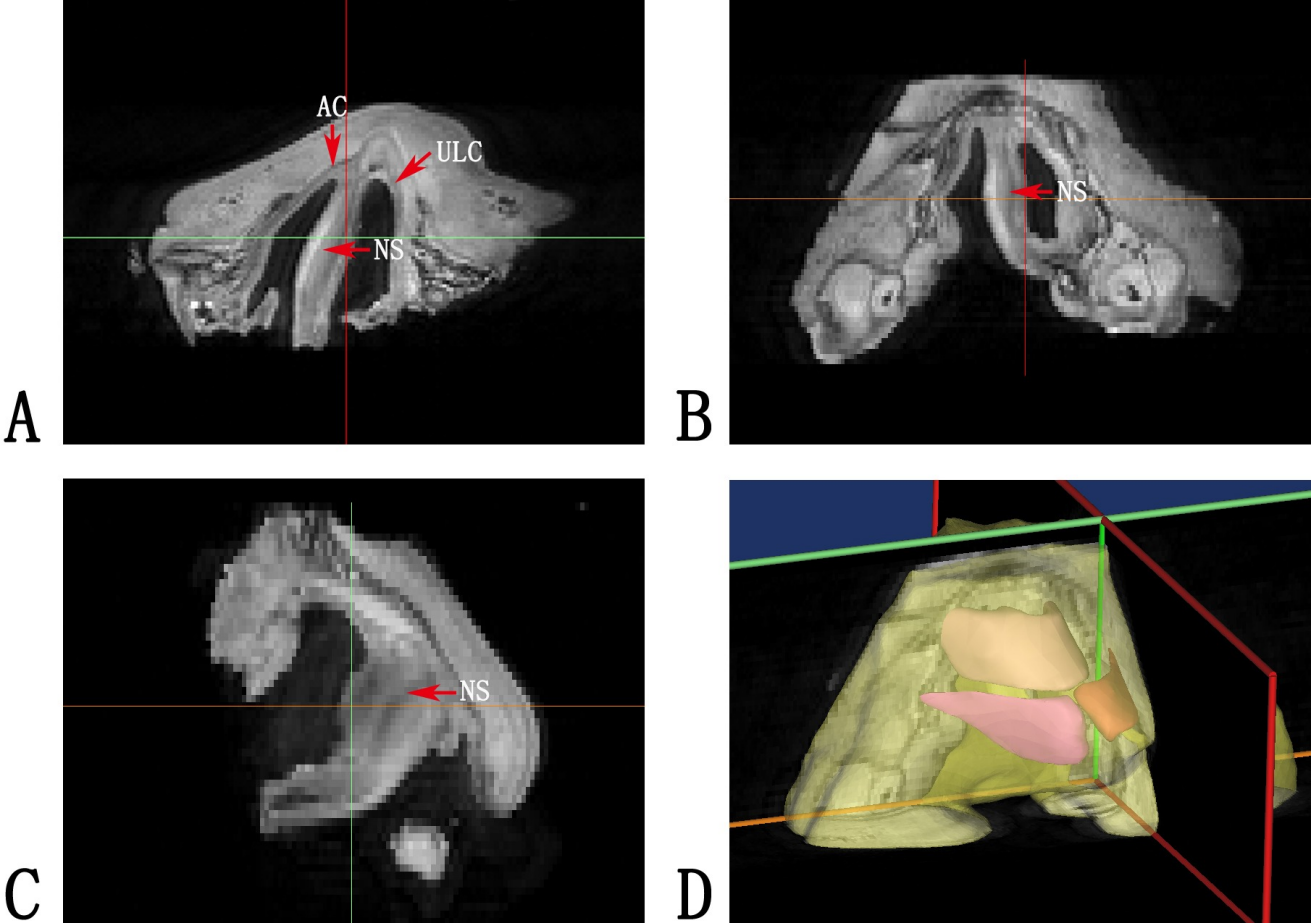
Micro-MRI imaging reconstruction. (A, B, C) Micro-MRI imaging of the fetal specimen. Red arrows indicated the position of cartilages, including the alar cartilages (AC), the upper lateral cartilages (ULC) and the nasal septum (NS). (D) Three dimensional reconstruction of the micro-MRI imaging.

Micro-MRI provided high-quality tissue definition for the nasolabial sample scanned. On the cross sections of the tomography, the nasal cartilage framework, including the alar cartilages and the upper lateral cartilages and the nasal septum, was clearly differentiated from the surrounding tissue. As a result, the three-dimensional reconstruction of the nasal cartilage provided an accurate morphological basis for further biomechanical analyses (Fig 2).

### Nasal deformation generated by simulations of rhinoplasty maneuvers

The nasal deformity among patients with unilateral cleft lip manifests three major characteristics, lateral displacement of the alar base on the cleft side, deviation of the columella to the noncleft side, and the collapse of the nasal tip. Correspondingly, surgical correction focused on three maneuvers, moving the alar base medially, straightening the columella, and buttressing the nasal tip, which were simulated by F1, F2, and F3 respectively.

**Fig 3:**
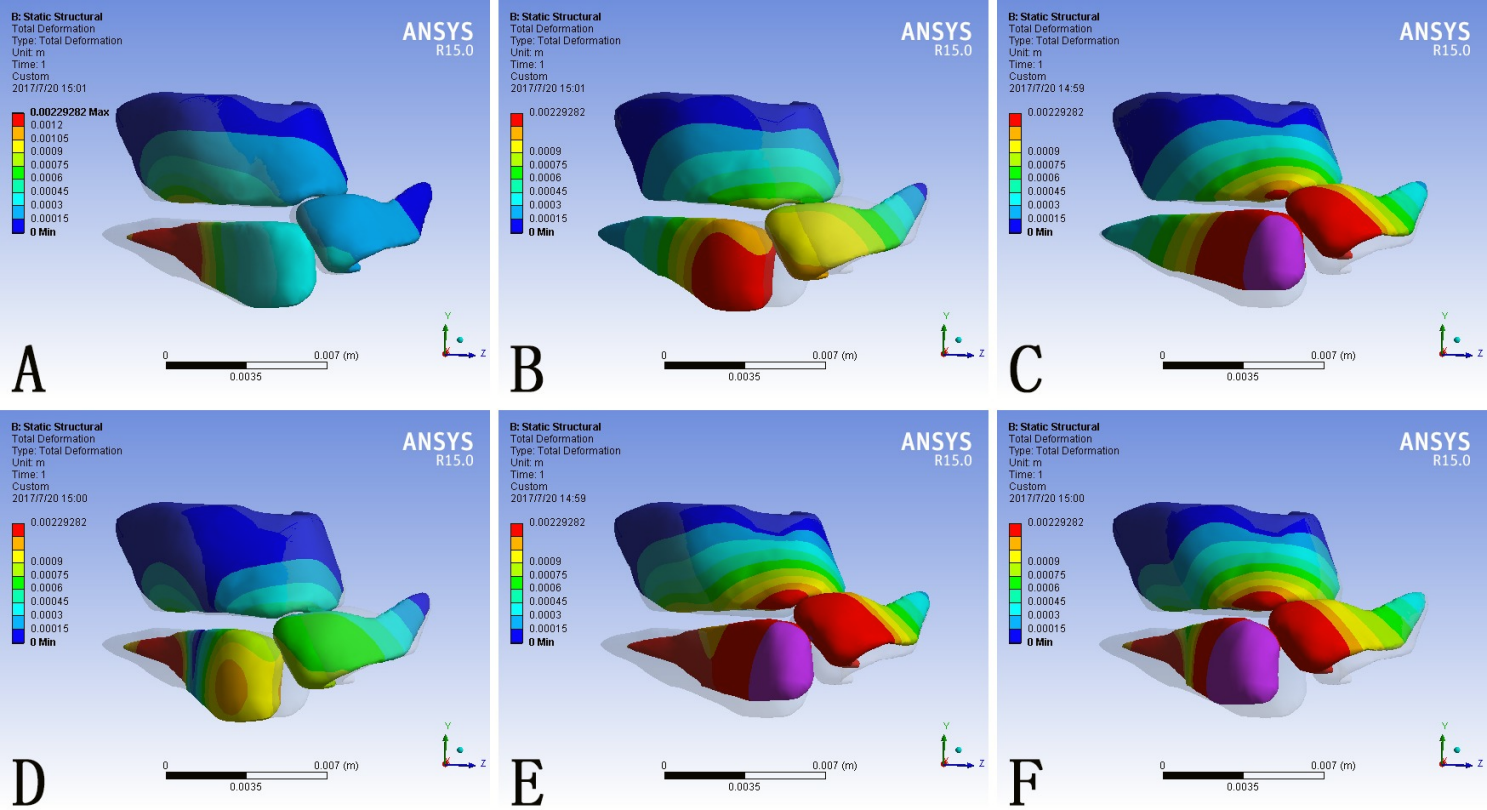
Total deformation of the nasal cartilage framework consequent to different force loadings. (A) F1 alone; (B) F2 alone; (C) F3 alone; (D) F1 plus F2; (E) F1 plus F3; (F) F1, F2 and F3 at the same time. Blue represented the fixed part of the model. The grey shadow represented the pre-simulation position of the model.

**Fig 4:**
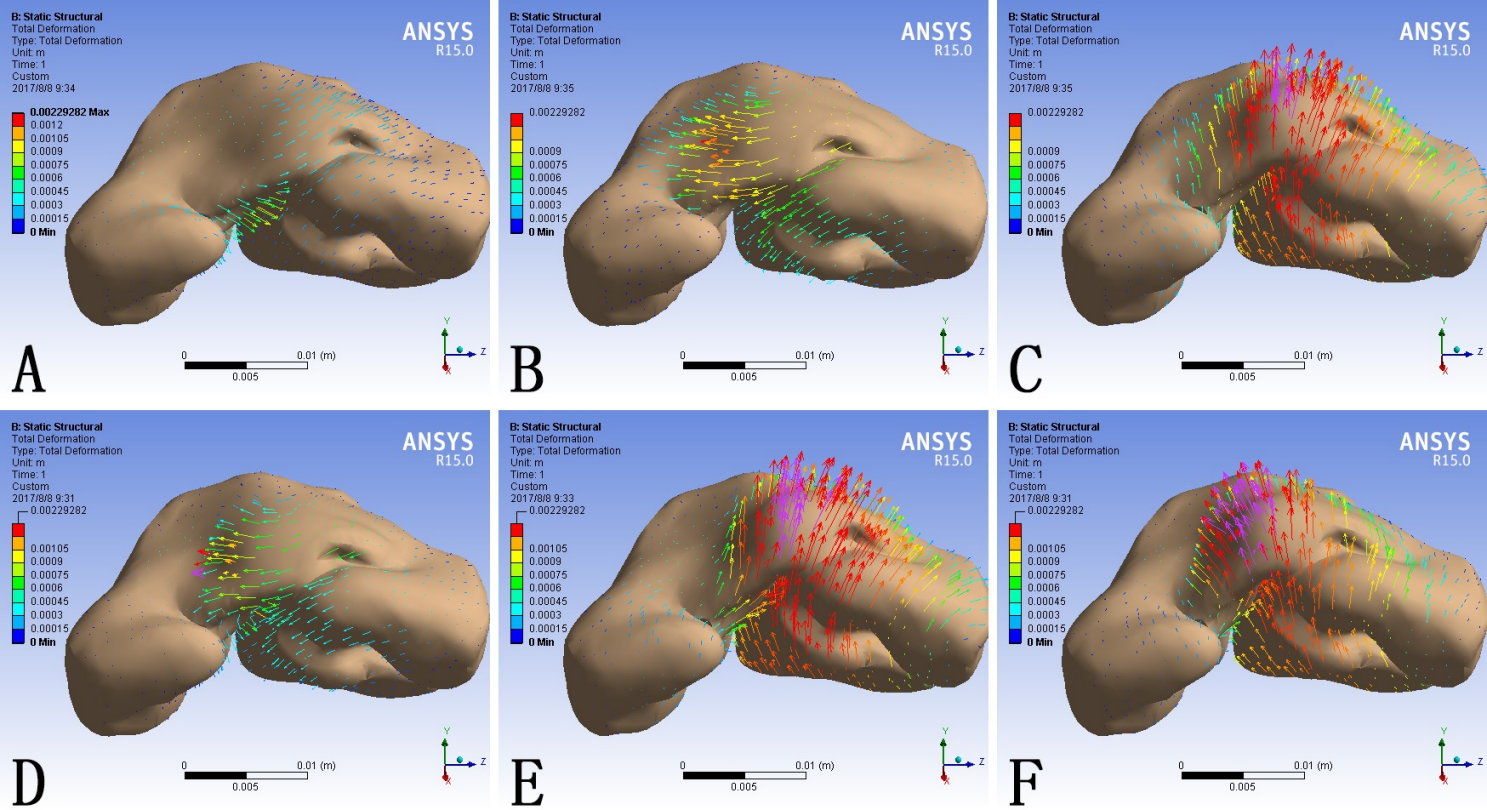
The deformation of the skin envelope consequent to different force loadings. (A) F1 alone; (B) F2 alone; (C) F3 alone; (D) F1 plus F2; (E) F1 plus F3; (F) F1, F2 and F3 at the same time. The length and direction of the arrow represented the value and direction of the deformation respectively.

The shape of the nose is decided by its underlying cartilage framework and similarly the cleft nasal deformity is caused by the displacement and hypoplasia of the cartliage. The total deformation of the model was scaled in millimeter and demonstrated as contour bands on the nasal cartilage framework (Fig 3) and color coded arrows on the skin envelope (Fig 4). The transparent shadow represented the original position of the model before force loading.

When loaded with F1 alone, the lateral crus of the alar cartilage at the cleft side moved towards the midline and the entire nasal framework moved towards the non-cleft side (Fig 3A). Correspondingly, the cleft side alar base and the columella base moved towards the non-cleft side (Fig 4A).

In contrary, when loaded with F2 alone, the cartilage framework, especially the alar cartilage at the cleft side, moved towards the cleft side (Fig 3B). The maximum deformation on the skin envelope was observed around the dome and the columella which moved towards the cleft side (Fig 4B).

When loaded with F3 alone, the entire cartilage framework moved anteriorly. The deformation reached its maximum at the intermediate crus on the cleft side and gradually attenuated towards bilateral lateral crura (Fig 3C). The nasal tip moved anteriorly, and the deformation extended to the alar bases at both sides (Fig 4C).

When loaded with F1 and F2 at the same time, the alar cartilage at the cleft side demonstrated counterclockwise rotation (Fig 3D). The alar base moved towards the midline and the base of the deviated columella moved towards the cleft side. Consequently the columella was straingthened and the cleft gap was narrowed. (Fig 4D).

When loaded with F1 and F3 at the same time, the cartilage framework moved anteriorly and the lateral crus at the cleft side moved towards the non-cleft side (Fig 3E). Correspondingly, the alar base at the cleft side moved towards the midline and the nasal tip moved anteriorly. In the absence of F2, the columella base also moved to the noncleft side, which further deviated the columella from the midline. (Fig 4E).

When loaded with F1, F2 and F3 at the same time, the alar cartilage at the cleft side underwent counterclockwise rotation and the whole cartilage framework moved anteriorly (Fig 3F). At the level of skin envelope, the alar base at the cleft side moved towards the midline, the base of the columella moved towards the cleft side, and the nasal tip moved anteriorly, which represented cleft gap narrowing, columella straightening, and nasal tip buttressing at the same time (Fig 4F).

What was notable is that, both on the cartilage framework and the skin envelope, each local force loading could incur morphological changes in the entire nose.

### Specific deformation and stress distribution on critical nasal landmarks

**Fig 5:**
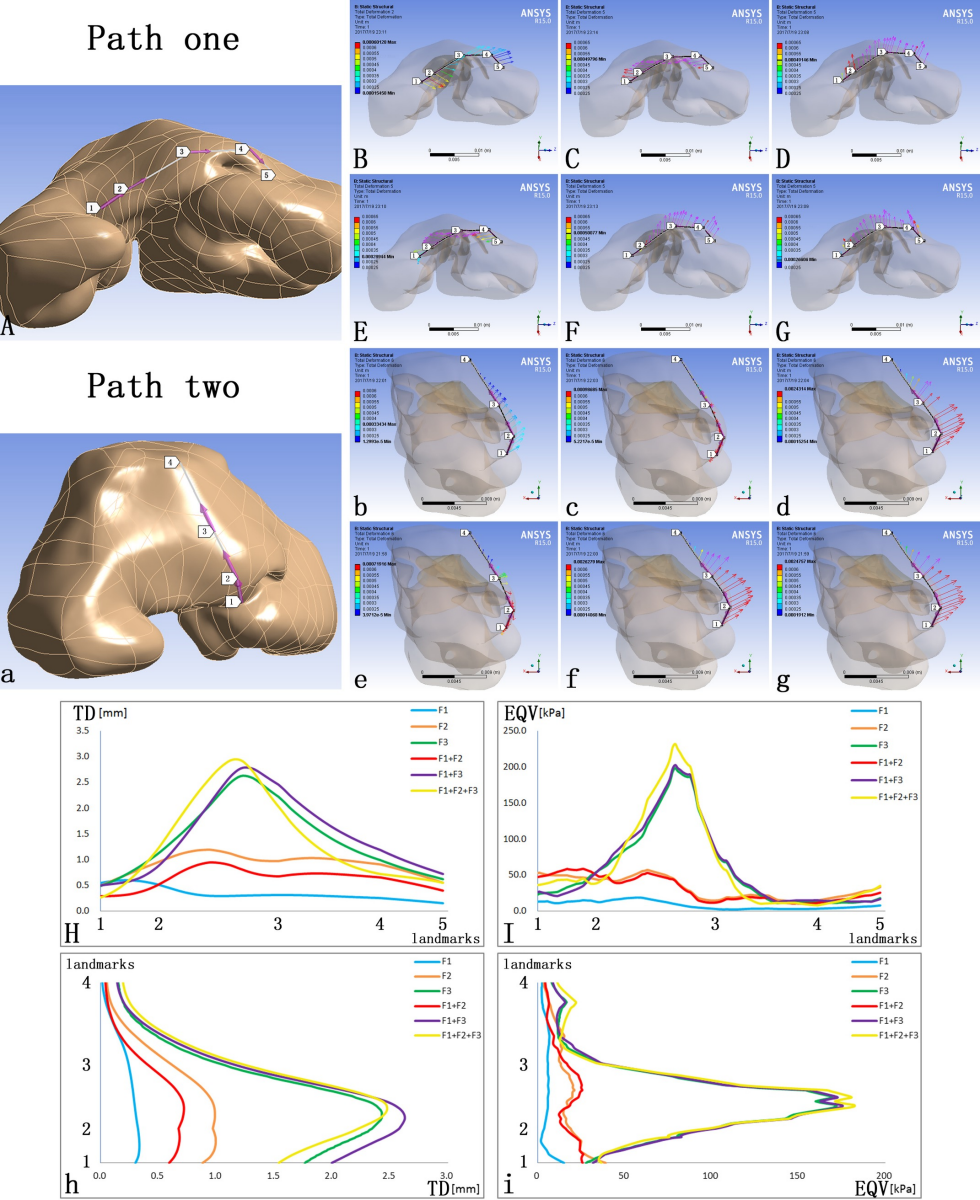
The TD and EQV at major landmarks on the skin envelope. (A) Path one was defined by the alar bases at both sides (Landmarks one, five), the alar domes at both sides (Landmark two, four) and the nasal tip (Landmark three); (a) Path two was defined by the columella base (Landmark one), the nasal tip (Landmark two), the dorsum (Landmark three) and the nasal radix (Landmark four); (B, C, D, E, F, G) The vectors of TD on Path one; (b, c, d, e, f, g) The vectors of TD on Path two; (H) The TD on Path one; (I) The EQV on Path one; (h) The TD on Path two; (i) The EQV on Path two.

Two paths on the cutaneous surface were defined to specify the TD and EQV at critical nasal landmarks. Path one was defined by the alar bases at both sides (Landmarks one, five), the alar domes at both sides (Landmark two, four) and the nasal tip (Landmark three) (Fig 5A). Path two was defined by the columella base (Landmark one), the nasal tip (Landmark two), the dorsum (Landmark three) and the nasal radix (Landmark four) (Fig 5A).

F1 alone moved Path one to the non-cleft side and Path two anteriorly (Fig 5Bb). F2 alone only moved Path one towards the cleft side with no significant anterior movement on Path two (Fig 5Cc). F3 alone moved both Path one and two anteriorly (Fig 5Dd). When loading F1 and F2 at the same time, the major consequence was to move Path one towards the cleft side except the alar base at the cleft side (Fig 5Ee). When loading F1 and F3 at the same time, Path one moved towards the non-cleft side and both paths moved anteriorly (Fig 5Ff). When loading F1, F2 and F3 at the same time, both paths moved anteriorly and no significant deviation occurred (Fig 5Gg).

When set at the same magnitude, F3 generated significantly larger TD than F1 or F2 alone on Path one and Path two (Fig 5Hh, Blue, Orange, Green). When loading F1 and F2, which were opposing to each other, at the same time, there was an offset effect in the generated TD (Fig 5Hh, Blue, Orange, Red). When loading F1 and F3 at the same time, the TD on Path one was the offset of the those caused by F1 and F3 alone at the cleft side and was the superposition of those on the non-cleft side. On Path two, the TD from the columella base to the tip was the superposition (Fig 5Hh, Blue, Green, Purple). When loading F1, F2 and F3 at the same time, the TD on Path one was larger at the cleft side and lower at the non-cleft side than loading F1 and F3 at the same time, and the TD on Path two was lower from the columella base to the nasal tip (Fig 5Hh, Purple, Yellow).When set at the same magnitude, F3 was associated with more significant morphological and stress changes in the nasal tip. The force loading of F3 alone, F1 plus F3, and F1, F2 plus F3 caused almost even value of stress on both paths (Fig 5Ii, Green, Purple, Yellow). Under the circumstances without F3, the addition of F2 would lead to higher stress (Fig 5Ii, Blue, Orange, Red).

## Discussion

Primary cleft lip rhinoplasty has been a big challenge to plastic surgeons in cleft care. Typical characteristics of unilateral cleft lip nasal deformities include collapsed alar dome, deviated nasal tip and skewed columella. Major intrinsic causes of cleft lip related nasal deformities are hypoplasia and displacement of the nasal cartilage framework [14–17]. Nowadays, various corrective procedures are practiced in different cleft centers, and the outcomes inevitably vary among them. Such a variation is not difficult to predict, since different techniques generated different tissue displacement and stress distribution within the nasal structure.

Early in 1998, Fisher and Mann designed a three-dimensional paper model of alar cartilages to analyze the structure of cleft lip nose [4]. These paper models were constructed basing on subjective experience. In 2005, finite-element scaling analysis was used to evaluate three-dimensional changes in nasal morphology in patients treated with presurgical nasoalveolar molding, and this analysis was sucessfully applied in the evaluation and prediction of nasal symmetry after presurgical orthopedics [5]. With the aid of the finite element analysis, both the cartilage framework and the skin envelope could be modeled with higher accuracy, and the deformation and the stress distribution could be quantified in detail. For the past decade, finite element analysis has already been successfully applied in the biomechanical analysis for rhinoplasty, covering nasal septum and the L-strut [6, 7, 18–21], the alar cartilages [22], the inverted-V deformity after surgery [8], the nasal tip [9–11, 23], and the nasal implants [24], and has been taken in the study of cleft lip nasal deformity [12].

In this study, FE modeling was used to capture the cleft lip nose structure and mimic the morphological and biomechanical consequences of different corrective procedures. Major surgical maneuvers were simplified and simulated by different force loadings: (1) medially directed force on the lateral crus (F1) simulated medial movement of alar base; (2) Laterally directed force on the medial crus (F2) simulated lateral movement of columella base; (3) Anteriorly directed force on the intermediate crus (F3) simulated nasal tip enhancement (Fig 1E and 1F). The deformation pattern predicted the immediate surgical outcome, whereas the distribution and magnitude of stress suggested the probability of relapse.

When loaded alone, the three forces generated disparate morphological changes. F1 mainly generated adduction of the alar base on the cleft side to the midline (Fig 4A) and also contributed to tip projection (Fig 5B). F2 led to relocation of the deviated columella and nasal tip to the midline(Fig 4Bb). F3 focused on enhancing the tip projection, which was more significant than that generated by F1 (Fig 5bd). Generally, these three forces corresponded with the three major deformities of unilateral cleft lip nose, the displacement of the alar base at the cleft side, the deviated columella and the loss of tip projection.

The combination of different force loadings generated obviously different outcomes. When loaded at the same time, F1 and F2 acted antagonistically. At the nasal tip, F2 counteracted the tip projection induced by F1 (Fig 5Ee). The deviated columella and nasal tip were relocated towards the midline, improving the symmetry of the nose (Fig 4D, Fig 5E). When loading F1 and F3 at the same time, the tip projection was further enhanced but the asymmetry became more evident. Neither the deviated columella base nor the deviated nasal tip was corrected (Fig 4E and Fig 5Ff). The combination of F1, F2 and F3 generated the most widespread morphological changes among the simulated circumstances. Both the collapsed nasal tip and the nasal asymmetry were relieved (Fig 4F and Fig 5Gg).

F3 generated the most intensive EQV when compared to F1 and F2 (Fig 5Ii). The EQV generated by F3 loading reached its maximum around the nasal tip and thus predicted a high relapse tendency in nasal tip collapse. Since the restoration of the tip projection was one of the major aims of primary cleft lip rhinoplasty, maneuvers simulated by F3 should be included in the surgical technique, but additional support to the nasal tip should be devised, nasal stent for example, to prevent relapse.

When F2 was loaded on top of F1-F3 combination, it further relieved nasal deviation without incurring significant increase in EQV (Fig 5Ii, Blue, Yellow). Since alar base adduction (represented by F1) and nasal tip enhancement (represented by F3) must be included in cleft lip repair, and columella base correction (represented by F2) would further enhance nasolabial morphology without increasing the probability of relapse, the soundest surgical technique should include maneuvers simulated by all three force loadings.

To be more specific, the three forces correspond to three particular surgical maneuvers. Medially directed force on the lateral crus (F1), indicating alar base adduction, could be achieved by suturing two alar bases together. This maneuver included reposition of orbicularis oris muscle to restore the alar flare. Laterally directed force on the medial crus (F2), indicating columella straightening, could be achieved by suturing the orbicularis oris muscle under columella base and alar base on the non-cleft side together to repair the oblique of columella. Anteriorly directed force on the intermediate crus (F3), indicating nasal tip elevation, could be achieved by the intercrural suture, which fixed the medial crura of bilateral alar cartilage at the nasal tip. Based on the results in this study, it would be more ideal to incorporate all of these three surgical maneuvers in correcting primary unilateral cleft lip nasal deformity.

Unlike previous studies, Micro-MRI was used to acquire the three dimensional information of nasal cartilage. While Micro-CT cannot differentiate cartilage from surrounding soft tissue [25], Micro-MRI, in contrast, can reveal the approximate location of the cartilages and offer a more accurate morphology of the cartilage framework for further modeling (Fig 2).

This study, however, bore the intrinsic limitations shared by all theoretical models. The mechanical properties of the CAD model were set in the elastic region rather than in the plastic region [8]. In addition, the accurate magnitude of force was arbitrarily defined. The reason for setting the magnitude at 5N is that the deformation generated on the skin envelope was close to the reality. The properties of human tissue are not homogeneous in reality, which would definitely influence the mechanical results. Moreover, the loading spot and direction of the force were difficult to set in accuracy and were chosen basing on experience. The bony structure was not taken into consideration, which was another limitation of our study because the morphology of bone could influence the changing mechanism of the overlap skin envelope and cartilages. Different bone or bony defect could lead to a bit different outcomes when loading forces on the cartilages. Finally, for feasibility considerations, surgical maneuvers were highly simplified into three force vectors on the alar cartilage.

## Conclusion

An accurate finite element model of cleft lip nasal deformity was established basing on the micro-MRI imaging. Major surgical correction maneuvers were successfully simulated in the form of force loading on the alar cartilage. The simulation suggested that alar base adduction, columella straightening, and nasal tip elevation should all be included in a competent cleft lip nose correction.

## References

[1] Vyas RM, Warren SM. Unilateral cleft lip repair. Clin Plast Surg. 2014;41(2):165–77. doi: 10.1016/j.cps.2013.12.009 . 24607186 10.1016/j.cps.2013.12.009

[2] Nolst Trenité GJ. Rhinoplasty: a practical guide to functional and aesthetic surgery of the nose Amsterdam; New York: Kugler Publications; 1993 x, 200 p. p.

[3] Gudis DA, Patel KG. Update on primary cleft lip rhinoplasty. Curr Opin Otolaryngol Head Neck Surg. 2014;22(4):260–6. doi: 10.1097/MOO.0000000000000066 . 24914745 10.1097/MOO.0000000000000066

[4] Fisher DM, Mann RJ. A model for the cleft lip nasal deformity. Plast Reconstr Surg. 1998;101(6):1448–56. . 9583472 10.1097/00006534-199805000-00003

[5] Singh GD, Levy-Bercowski D, Santiago PE. Three-dimensional nasal changes following nasoalveolar molding in patients with unilateral cleft lip and palate: geometric morphometrics. Cleft Palate Craniofac J. 2005;42(4):403–9. doi: 10.1597/04-063.1 . 16001922 10.1597/04-063.1

[6] Lee JS, Lee DC, Ha DH, Kim SW, Cho DW. Redefining the septal L-strut in septal surgery. PLoS One. 2015;10(3):e0119996 doi: 10.1371/journal.pone.0119996 ; PubMed Central PMCID: PMCPMC4372341. 25803842 10.1371/journal.pone.0119996 PMC4372341

[7] Lee JS, Lee DC, Ha DH, Kim SW, Cho DW. Redefining the Septal L-Strut to Prevent Collapse. PLoS One. 2016;11(4):e0153056 doi: 10.1371/journal.pone.0153056 ; PubMed Central PMCID: PMCPMC4830535. 27073993 10.1371/journal.pone.0153056 PMC4830535

[8] Tjoa T, Manuel CT, Leary RP, Harb R, Protsenko DE, Wong BJ. A Finite Element Model to Simulate Formation of the Inverted-V Deformity. JAMA Facial Plast Surg. 2016;18(2):136–43. doi: 10.1001/jamafacial.2015.1954 . 26720757 10.1001/jamafacial.2015.1954 PMC5828020

[9] Manuel CT, Harb R, Badran A, Ho D, Wong BJ. Finite Element Model and Validation of Nasal Tip Deformation. Ann Biomed Eng. 2016 doi: 10.1007/s10439-016-1729-9 . 27633018 10.1007/s10439-016-1729-9 PMC5332342

[10] Shamouelian D, Leary RP, Manuel CT, Harb R, Protsenko DE, Wong BJ. Rethinking nasal tip support: a finite element analysis. Laryngoscope. 2015;125(2):326–30. doi: 10.1002/lary.24845 ; PubMed Central PMCID: PMCPMC4304991. 25130506 10.1002/lary.24845 PMC4304991

[11] Leary RP, Manuel CT, Shamouelian D, Protsenko DE, Wong BJ. Finite Element Model Analysis of Cephalic Trim on Nasal Tip Stability. JAMA Facial Plast Surg. 2015;17(6):413–20. doi: 10.1001/jamafacial.2015.0941 . 26427012 10.1001/jamafacial.2015.0941 PMC5847269

[12] Huang H, Li Y, Luo X, Cheng X, Shi B, Li J. Mechanical analyses of critical surgical maneuvers in the correction of cleft lip nasal deformity. PLoS One. 2018;13(4):e0195583 doi: 10.1371/journal.pone.0195583 ; PubMed Central PMCID: PMCPMC5898757. 29652906 10.1371/journal.pone.0195583 PMC5898757

[13] Luo X, Yang B, Sheng L, Chen J, Li H, Xie L, et al CAD based design sensitivity analysis and shape optimization of scaffolds for bio-root regeneration in swine. Biomaterials. 2015;57:59–72. doi: 10.1016/j.biomaterials.2015.03.062 . 25913251 10.1016/j.biomaterials.2015.03.062

[14] Wolfe SA. A pastiche for the cleft lip nose. Plast Reconstr Surg. 2004;114(1):1–9. . 15220558 10.1097/01.prs.0000130416.86351.24

[15] McComb HK, Coghlan BA. Primary repair of the unilateral cleft lip nose: completion of a longitudinal study. Cleft Palate Craniofac J. 1996;33(1):23–30; discussion -1. doi: 10.1597/1545-1569_1996_033_0023_protuc_2.3.co_2 . 8849855 10.1597/1545-1569_1996_033_0023_protuc_2.3.co_2

[16] McComb H. Primary correction of unilateral cleft lip nasal deformity: a 10-year review. Plast Reconstr Surg. 1985;75(6):791–9. . 4001197 10.1097/00006534-198506000-00003

[17] Huffman WC, Lierle DM. Studies on the pathologic anatomy of the unilateral harelip nose. Plast Reconstr Surg (1946). 1949;4(3):225–34. . 18131353 10.1097/00006534-194905000-00001

[18] Lee SJ, Liong K, Tse KM, Lee HP. Biomechanics of the deformity of septal L-Struts. Laryngoscope. 2010;120(8):1508–15. doi: 10.1002/lary.20976 . 20564665 10.1002/lary.20976

[19] Liong K, Lee SJ, Lee HP. Preliminary Deformational Studies on a Finite Element Model of the Nasal Septum Reveals Key Areas for Septal Realignment and Reconstruction. J Med Eng. 2013;2013:250274 doi: 10.1155/2013/250274 ; PubMed Central PMCID: PMCPMC4782633. 27006910 10.1155/2013/250274 PMC4782633

[20] Lee SJ, Liong K, Lee HP. Deformation of nasal septum during nasal trauma. Laryngoscope. 2010;120(10):1931–9. doi: 10.1002/lary.21072 . 20824645 10.1002/lary.21072

[21] Mau T, Mau ST, Kim DW. Cadaveric and engineering analysis of the septal L-strut. Laryngoscope. 2007;117(11):1902–6. doi: 10.1097/MLG.0b013e3181255ec4 . 17721403 10.1097/MLG.0b013e3181255ec4

[22] Oliaei S, Manuel C, Protsenko D, Hamamoto A, Chark D, Wong B. Mechanical analysis of the effects of cephalic trim on lower lateral cartilage stability. Arch Facial Plast Surg. 2012;14(1):27–30. doi: 10.1001/archfacial.2011.1354 ; PubMed Central PMCID: PMCPMC4131858. 22250265 10.1001/archfacial.2011.1354 PMC4131858

[23] Manuel CT, Leary R, Protsenko DE, Wong BJ. Nasal tip support: a finite element analysis of the role of the caudal septum during tip depression. Laryngoscope. 2014;124(3):649–54. doi: 10.1002/lary.24321 ; PubMed Central PMCID: PMCPMC4364034. 23878007 10.1002/lary.24321 PMC4364034

[24] Jung JW, Park JH, Hong JM, Kang HW, Cho DW. Octahedron pore architecture to enhance flexibility of nasal implant-shaped scaffold for rhinoplasty. Int J Precis Eng Man. 2014;15(12):2611–6. doi: 10.1007/s12541-014-0634-0 PubMed PMID: WOS:000346166800017.

[25] Wu J, Yin N. Detailed Anatomy of the Nasolabial Muscle in Human Fetuses as Determined by Micro-CT Combined With Iodine Staining. Ann Plast Surg. 2016;76(1):111–6. doi: 10.1097/SAP.0000000000000219 . 25003453 10.1097/SAP.0000000000000219

